# A Thermoresponsive and Magnetic Colloid for 3D Cell Expansion and Reconfiguration

**DOI:** 10.1002/adma.201403626

**Published:** 2014-11-29

**Authors:** Aram Saeed, Nora Francini, Lisa White, James Dixon, Toby Gould, Hassan Rashidi, Racha Cheikh Al Ghanami, Veronika Hruschka, Heinz Redl, Brian R Saunders, Cameron Alexander, Kevin M Shakesheff

**Affiliations:** 1 School of Pharmacy, University of East AngliaNorwich, NR4 7TJ, UK; 2 School of Pharmacy, University of NottinghamNottingham, NG7 2RD, UK; 3 Ludwig Boltzmann Institute for Experimental and Clinical TraumatologyVienna, Austria; 4 School of Materials, University of ManchesterManchester, M13 9PL, UK

The ability to harness cells as therapeutic products remains a scientific challenge of major significance.^1^ Recent studies and clinical trials have shown the potential of cell-based therapies for the treatment of a range of conditions including cardiovascular,^2^ neurologic,^3^ and autoimmune diseases.^4^ However, to transfer cell therapies from the research laboratory toward practical application where cells are needed in high volumes, new manufacturing tools are required. A variety of techniques have been used to grow cells in vitro with increasing emphasis on 3D matrices in order to overcome the inherent difficulties associated with 2D culture.^5^ In comparison to the standard 2D systems, 3D matrices provide higher surface areas to support growth to larger cell volumes and produce microenvironments which better mimic in vivo systems.^6^,^7^ Recent advances include the use of 3D printing technologies to create cell patterns in a controlled fashion^8^–^11^ and there are several marketed products to allow formation of 3D cell supports such as Matrigel, Alvetex, QGel, and Biomerix. Although these matrices are widely used in research settings, the potential for their use in cell manufacturing at high cell volumes has yet to be reported. Moreover, the fixed internal structures of some of these materials pose technical challenges, making it difficult to seed cells in controllable locations or to passage cells at specified time points, as well as to harvest the cells efficiently after expansion. For example, cells are usually seeded into support matrices under static or low-flow conditions that rely on diffusional processes and weak adhesion forces, resulting in inhomogeneous cell localization and growth.^12^ Cell seeding under dynamic flow conditions is possible using industrial bioreactors with built-in perfusion systems,^13^ but this is more problematic when manipulating the small amounts of primary cells available from donors. Other technical challenges for cell manufacture include the requirement to use proteolytic enzymes to detach cells from support materials during passaging or after cell expansion. Enzymes such as trypsin can cause cleavage of membrane proteins and growth factor receptors thus altering the proteomic profile in mammalian cells.^14^–^16^ There is accordingly a need for new materials that can support expansion of clinically relevant cell types, which can be easily manipulated to allow cell recovery, and which can be reconfigured on demand to enable patterning of varying cell types. We report here a dual functional colloidal gel system, which combines the advantages of allowing homogeneous cell seeding, eliminates the need to use proteolytic enzymes during passaging, and enables placement of cells in discrete environments which can be patterned magnetically. Furthermore, the methodology is inherently scalable, allowing culture in volumes ranging from those appropriate to primary cells for single patient use through to stem cell manufacture.

Approaches to fabricate 3D cell support matrices include chemical cross-linking, solvent casting, particulate leaching, gas forming, and phase-separation emulsion or freeze-drying techniques.^17^ Although each method has certain advantages, there is no single technique that can be used to produce material assemblies that address all the fundamental problems linked to cell seeding, passaging, and harvesting. The conflicting requirements of a matrix that is mechanically strong enough to support cell growth and manipulation, yet easily dismantled to allow cell recovery rule out the use of a single material type. However, stimuli-responsive polymers offer a means by which materials properties can be switched,^18^–^20^ and in previous papers we have shown that it is possible to grow fibroblast cells in a reversibly assembling colloidal gel, utilizing a thermoresponsive polymer to control aggregation and disassembly of a matrix around a cell population.^21^,^22^ Nevertheless, the separation of cells from these gels was not efficient enough over repeated passages for stem cell expansion, and there were no means to move one region of a cell-containing gel next to another of a different cell type without mechanical micro­manipulation. We now show how the combination of an amphiphilic thermoresponsive material with polymer microparticles containing superparamagnetic cores can be used to create a 3D support matrix that allows not only efficient stem cell expansion over several passages but also cell pattern formation as a first step to tissue-type biological organization. The material can be rapidly and reversibly transformed from a dispersed suspension into a solid matrix by temperature elevation only, thus enabling the seeding of cells in a homogenous fashion, which can be quickly disassembled to release cells by a small reduction in temperature, and then the cells can be simply separated from the colloidal particles by a magnetic field. We further demonstrate that by combining the reversible assembly with magnetic field manipulation, the colloidal gel particles can be used to pattern co-cultured cells into a variety of architectures and that the 3D matrix can be used to expand stem cells in an efficient manner while maintaining the desired stem cell lineage during multiple cell growth and harvesting cycles.

The first part of the strategy involved the synthesis of a thermoresponsive polymer and magnetic microparticles, to be mixed together to form the 3D scaffold. We chose to fabricate the microparticles using polystyrene, as this material is already widely used in cell culture assays. Dispersion polymerizations of styrene (PS) with divinylbenzene (4 mol%) generated lightly cross-linked PS.^23^ Embedding of iron oxide (Fe_3_O_4_) in the surface of the microparticles was carried out by further polymerizations of styrene, in the presence of Fe_3_O_4_ powder stirred with the particles, and then by a final polymerization of styrene to cap the microparticles. We targeted the sizes of the magnetic polystyrene microparticles (MPSMs) to be larger than 1 μm to avoid possible internalization by cells. Scanning electron microscopy (SEM) images of the resultant Fe_3_O_4_-loaded polymers showed spherical microparticles with diameters in the range of 2–2.5 μm (Figure S1, Supporting Information). Thermogravimetric analysis showed an approximate iron oxide content of 15% (w/v) (Figure S2, Supporting Information).

For the thermoresponsive polymer, a dodecanethiol chain transfer agent was used in conventional free radical polymerization of 2-(2-methoxyethoxy) ethyl methacrylate (MEO_2_MA).^24^ Proton nuclear magnetic resonance (^1^H NMR) and gel permeation chromatography (GPC) confirmed the structure of the polymer (Figure S3, Supporting Information), with average number molecular weight of (*M*_n_ ≈ 20 kDa). The provision of a hydrophobic dodecyl chain end was designed to enhance adsorption of the otherwise hydrophilic poly(MEO_2_MA) to the hydrophobic MPSMs. To form the temperature reversible 3D colloid, the dodecyl-terminated polymer (DD-pMEO_2_MA) was physically mixed with MPSMs at mass ratios which enabled the colloids to be handled with ease at room temperature and yet be self-supportive gels within a few seconds of heating to 37 °C (**Figure** **1**).

**Figure 1:**
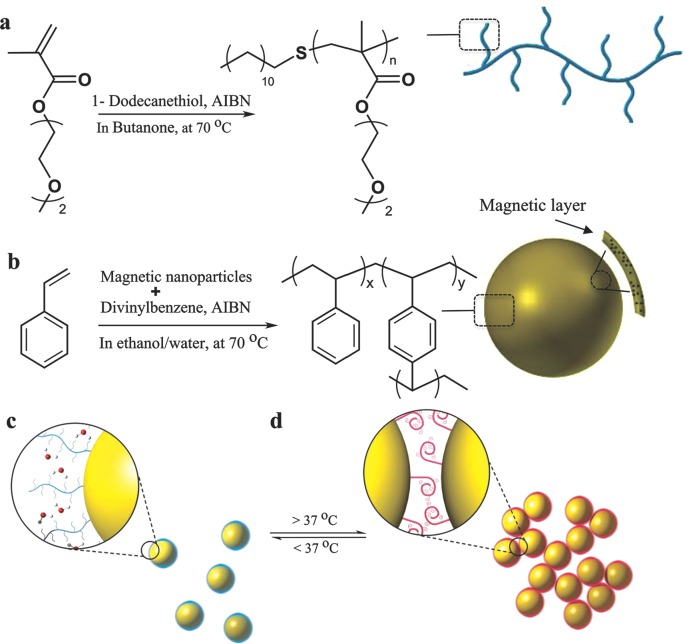
A schematic of the thermoreversible and magnetic 3D matrix synthesis route. a, b) Synthesis of the thermoresponsive and magnetic microparticles a) dodecanethiol chain transfer agent was used in the free radical polymerization of 2-(2-methoxyethoxy) ethyl methacrylate (MEO_2_MA) monomers to make thermoresponsive (DD-pMEO_2_MA) polymer. b) Dispersion polymerization of styrene with divinylbenzene cross-linker to generate magnetic polystyrene microparticles (MPSMs), which were then followed by embedding of iron oxide (Fe_3_O_4_) nanoparticles in the surface of the microparticles by further polymerization in the presence of styrene monomers. c, d) Showing self-assembly of the colloidal gel in which c) the matrix exists in free-flowing suspension below 37 °C, but d) reversibly solidify to form 3D porous matrix upon heating to 37 °C, causing the DD-pMEO_2_MA chains to collapse on the surface of MPSM.

Following the synthesis steps, the ability to seed and culture cells within a 3D matrix formed by the self-assembling colloidal gel was evaluated. Ratios of MPSM (25% w/v in culture media) and (DD-pMEO_2_MA) (4% w/v in culture media) were found to provide the best balance of mixing and rapid reversible gelation properties. We first seeded immortalized bone marrow-derived human mesenchymal stem cells (hMSCs), which were genetically modified to express green fluorescent protein (GFP hMSCs), in order to visualize the localization of the cells within the 3D gel and also to monitor cell proliferation qualitatively. Two methods were used to seed the cells onto the matrix, in order to simulate use by individuals in a research setting, or for a case in which automated systems for high-throughput manufacture would be used. For the first method, a suspension containing approximately 2 × 10^5^ GFP hMSCs was gently mixed with a known volume of the matrix in liquid state, followed by dropwise addition into prewarmed cell culture media (37 °C), (**Figure** **2**a, top and bottom). In the second method, the mixture of cells and particles was first added onto a dry slide (coated with polytetrafluoroethylene (PTFE) to produce a superhydrophobic surface), to form spherical droplets, and allowed to set in an incubator at 37 °C for 1 min before transferring into prewarmed media. As apparent from 2Figure, these methods enabled the size of the cell-support matrices to be readily manipulated by controlling the initial volumes, for example 10, 20, 50, and 100 μL (2Figure b, top and bottom). The schematic representations (2Figure c, top) were confirmed by the fluorescent images showing a homogeneous distribution of cells throughout the matrices (2Figure c, bottom).

**Figure 2:**
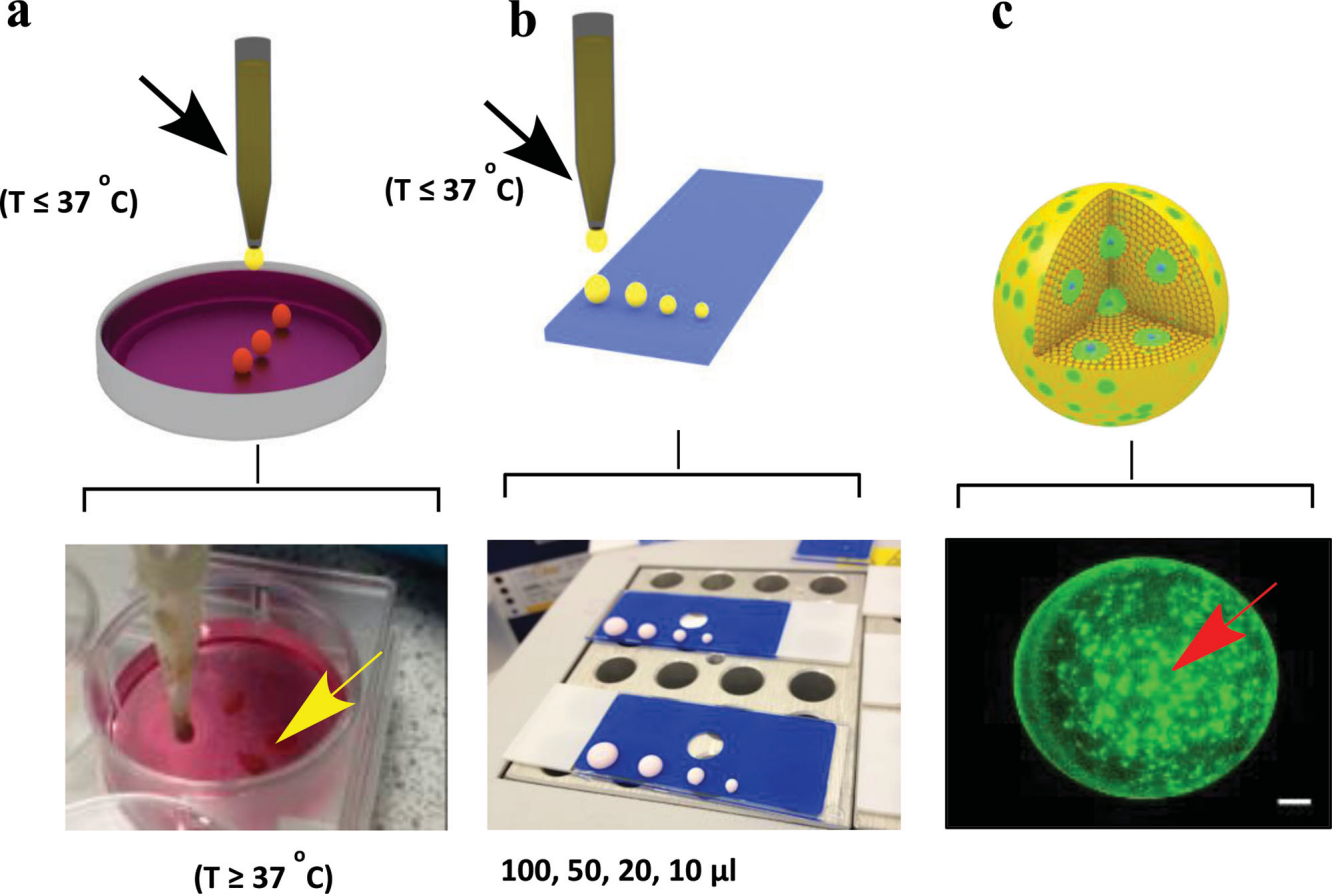
The generation of 3D matrix seeded with cells. a) Suspension of cells and matrix in liquid state (below 37 °C) mixed and added dropwise into a prewarmed cell culture media (37 °C); b) suspension of cells and matrix in liquid state added dropwise on a dry slide (coated with polytetrafluoroethylene to produce superhydrophobic surface), forming droplet-like cell-seeded matrix, which was then allowed to set in incubator at 37 °C for 1 min before transferring into prewarmed media. c) The size of cell-seeded 3D matrix was readily manipulated by controlling the initial volumes of 10, 20, 50, and 100-μL depositions, which resulted in c) homogeneous cell distribution of GFP-labeled ihMSCs within the matrix. Scale bar = 100 μm.

Having established the ability to seed cells uniformly into the 3D matrices, we investigated the use of the colloidal gel to support repeated expansion of cells. After seeding 5 × 10^4^ cells into 50 μL of colloidal gel (formed on the PTFE surfaces), cell growth was monitored by recording fluorescence images up to day 16 of incubation, using rhodamin-labeled MPSMs to provide contrast against the GFP-expressing cells. **Figure** **3** indicates that GFP hMSCs expanded progressively as compared to initial cell seeding. Furthermore, to investigate whether the cells grew equally throughout the outer and the core region of the gel, the matrices were sectioned after day 16 (Figure S4, Supporting Information). The fluorescence images from different sections of the matrices revealed uniform cells growth, most likely due to the large pores created by the particles which allows for rapid provision of oxygen and nutrient or removal of cellular wastes in and out of the matrix.

**Figure 3:**
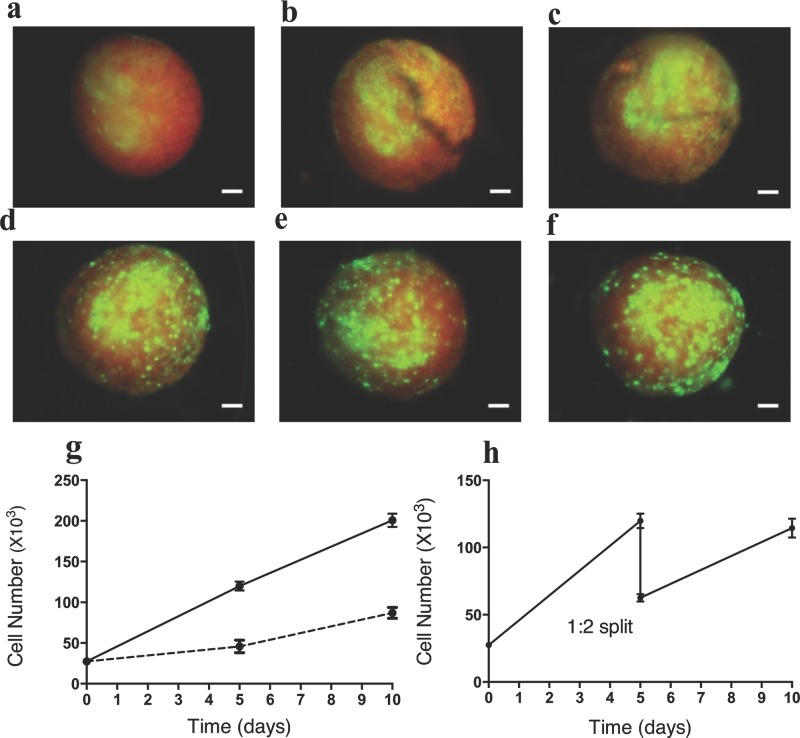
The expansion of hMSCs in 3D matrix and enzyme-free passaging. a–f) Cells expansion of GFP-tagged hMSCs seeded within rhodamin-labeled 3D matrix, fluorescent images were recorded on a) day 1, b) day 3, c) day 5, d) day 10, e) day 13, and f) day 16, showing progressive increase in cell proliferation, compared to initial cell seeding at day 1. g) Proliferation of unlabeled hMSCs as monitored by standard PrestoBlue assay up to day 10, cells proliferated on 3D matrix (solid line) compared to tissue-cultured plastic (dashed line) for the same initial cell seeding density and incubation time. h) Proliferated cells on 3D matrix were passaged at day 5 (without using any enzymes) by liquefying the “parent” matrix, followed by addition of equal volume of new matrix (without cells) into the parent matrix and subsequently formed two new “daughter” matrices (formed on PTFE slides). The daughter matrices were further incubated to day 10; at this point, the total cell numbers in daughter matrices were equal to the patent matrix before passaging. Error bars are ± standard deviations. Scale bar = 100 μm.

Having demonstrated cell proliferation using GFP-labeled MSCs, we sought to expand the investigation to use hMSCs. The same method to seed hMSCs was used as mentioned above; however, in this case cell expansion was monitored using a standard method (PrestoBlue). It can be seen from Figure 3g that the hMSCs (25 × 10^3^ cells in 25-μL matrix) showed a linear proliferation profile up to day 10, at which point the experiment was terminated to draw a comparison with HMSCs cultured on 2D plastic surfaces. Equal amounts of cells were initially seeded on both the 3D matrices and 2D culture plastic; however, the cell growth on the 2D was comparably lower than in the 3D gel. As expected, the total cell numbers on the 3D matrices were higher compared to cells grown on the 2D plastic. For example, the total number of cells grown on the 3D matrices was in the range of 119 × 10^3^ (4.7-fold increase) and 200 × 10^3^ (eight-fold increase) at day 5 and day 10, respectively. However, for the same seeding density on the 2D culture plastic, the total cell numbers were 45 × 10^3^ (1.8-fold increase) and 87 × 10^3^ (3.48-fold increase) at day 5 and day 10, respectively. These data suggested that the 3D colloidal gel not only supported proliferation of stem cells but also generated a high cell volume compared to classical 2D plastics, thus establishing a key practical advantage of using a 3D matrix system.

A potentially significant second advantage of a reversibly assembling colloidal gel is the ability to passage cells without using a trypsinization process or other biochemical cell detachment method, a required step to detach cells from most substrates including current 2D and 3D matrices. As indicated earlier, the colloidal 3D matrices can reversibly be transformed from liquid-suspension to solid-like matrices through a simple and also rapid temperature modulation. Cell growth and passaging experiments were carried out using the same cell seeding and incubation process as before; however, the HMSCs were passaged at day 5 of incubation, and were then further incubated for an additional 5 days. As shown in Figure 3h, the total number of cells measured at day 5 was 119 × 10^3^, and these cells were then passaged. It is important to note that cells did not need to be separated from the matrices at the passaging point. The passaging step was performed in a sequential manner. First, the nutrient media were removed from the cell culture “parent matrix” by gentle aspiration. Then the parent matrix gel was returned to the flowable colloidal suspension state by cooling the system to room temperature. An aliquot of freshly prepared particle suspension (containing no cells), equal in volume to the parent matrix, was added and the whole suspension was gently mixed. Following this addition, the resulting cell/particle-mixed suspension was split into two equal volume aliquots or “daughter matrices”, such that the concentration of the cells in the suspension was half that of the original parent matrix. Finally, the mixed matrices, still in liquid phase, were split into two equal volumes to create daughter matrices, and these were further incubated as described above.

For application to stem cell manufacture, expansion on a support matrix must maintain the desired phenotypic signature, characterized, for example, by the specific immunophenotypic surface markers expressed by MSCs. Accordingly, the surface markers CD90, CD73, and CD105 were used for positive hMSCs identity and CD45, CD34, CD19, CD11b, and HLA-DR were used as negative markers.^25^ Cells were analyzed before and after 5-days incubation in the 3D gels, using standard flow cytometry protocols. Analysis indicated (Table S1, Supporting Information) that the surface markers remained unchanged before and after inoculation on 2D tissue culture plastic (TCP) and the 3D matrix, and cells were adherent to standard culture plastic. For both the 2D TCP and the 3D matrix, as well as both pre- and postinoculation, >99% of the cell population were positive for CD90, CD73, and CD105, while the numbers of cells displaying the markers CD45, CD34, CD19, CD11b, and HLA-DRA were <2%. These results suggest that the 3D matrix may be advantageous for the expansion of clinically important cell type such as MSCs.

In order to accomplish the complete process from cell seeding to harvesting, we examined whether the expanded HMSCs could be efficiently separated from the matrices to recover cell populations in high numbers at the end point. The separation and recovery process is as illustrated in **Figure** **4**a, with sequentially recorded digital images in Figure 4b, showing that the matrix transformed from a gel to a liquefied suspension from which the cells were separated by applying an external magnetic bar in close proximity. The total recovery of hMSCs was also quantified at days 5, 10, and 15 using the PrestoBlue assay (Figure 4c). This was an essential step not only to quantify the cells but also to ensure the cells retained full viability. These data demonstrated a consistently high cell recovery of 93.5%, 95.6%, and 92.1% from the matrices at days 5, 10, and 15, respectively. By contrast, poor cell recoveries were observed when a conventional centrifugation process was applied to the gel (data not shown). These data indicated the critical importance of combining the magnetic core component of the particles with the thermoreversible shell; in this way, the colloidal gel could be easily separated from the cells using a low shear stress magnetic separation step after cooling, thus enabling recovery of cells rapidly and in high volumes.

**Figure 4:**
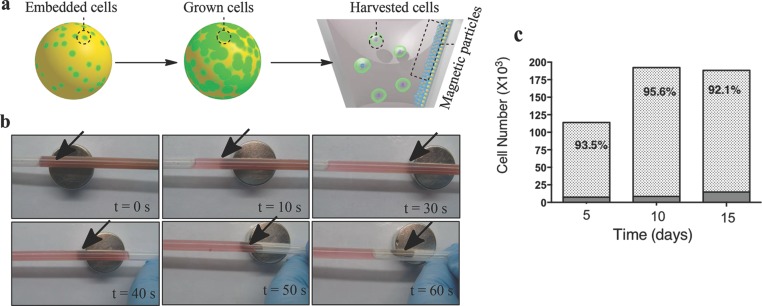
The recovery of cells from 3D matrix by magnetic extraction. a) Illustration of cell recovery steps by magnetic extraction, b) sequentially recorded digital images showing liquefied cells and matrix mixture separated by applying external magnetic bar in close proximity (arrow denotes separated MPSM). c) Total recovery of hMSCs at days 5, 10, and 15 as quantified by PrestoBlue assay, which was also used to show that the cells retained viability. Cell recovery of 93.5%, 95.6%, and 92.1% was achieved at days 5, 10, and 15, respectively.

Having established some practical advantages for stem cell expansion, we investigated the possibilities for patterning cells within discrete zones in 3D, as a first step to tissue modeling in vitro. Cultured 3T3 fibroblasts expressing RFP (red) and GFP (green) were incorporated into the 3D matrices, at cell densities of 1 × 10^6^ per 20 μL of the colloidal particle suspension. By moving a magnetic bar underneath free flowing bead suspensions in culture media, followed by in situ gelation, it was possible to form patterned gels in layers in partial mimicry of the organization of cells in normal tissue. As shown in **Figure** **5**a (left and right), patterns were generated comprising: i) multi­ple regions of GFP 3T3 cells with a single RFP 3T3-seeded matrix in the center (left) and ii) a two-layered pattern with alternating zones of RFP and GFP-expressing fibroblasts (right). We also investigated the reconfiguration of cell patterns, using the magnetic field as a noninvasive method, and were able to demonstrate dynamic mixing of the cells after culture. RFP 3T3 and GFP 3T3 cells were incorporated into magnetic and nonmagnetic matrices, respectively. We then aligned the matrices together and applied repeated magnetic fields bar by sequential movement of a magnetic bar from side to side. The fluorescence microscopy images revealed a progressive reconfiguration of the matrices from discrete colored zones into a single region containing both cell types. Cell viability was unaffected by the reconfiguration process as determined by PrestoBlue assay (data not shown).

**Figure 5:**
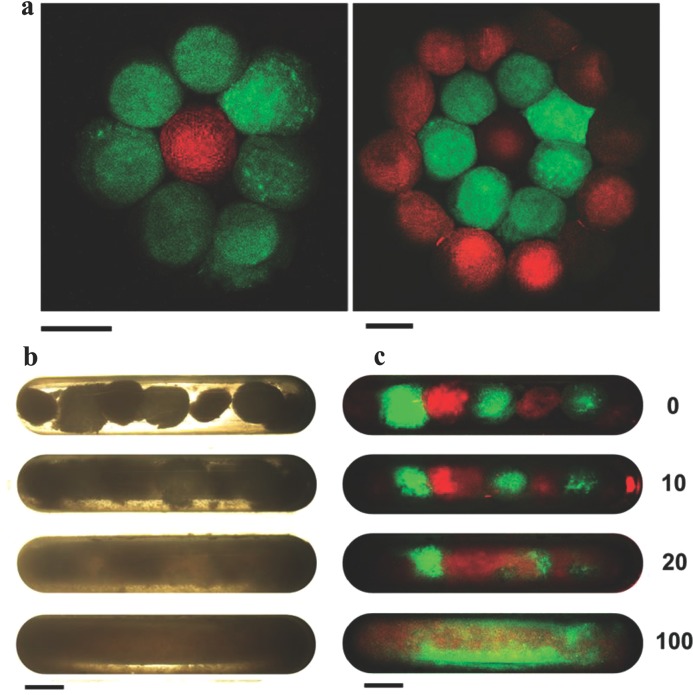
The cell patterns and reconfiguration in 3D. Cultured 3T3 fibroblasts expressing RFP (red) and GFP (green) were seeded into the 3D matrices, at 1 × 10^6^ cells per 20 μL of the matrix. a) Fluorescent images showing cell patterns in 3D formed as multiple regions of GFP 3T3-seeded matrices with a single RFP 3T3-seeded matrix in the center (left), (right) two-layered patterns with alternating zones of RFP- and GFP 3T3-seeded matrices. b,c) Reconfiguration of cells patterns shown in b) bright field and c) fluorescent images, in which reconfiguration of the cell patterns was demonstrated by seeding RFP- and GFP-3T3 cells into magnetic and nonmagnetic 3D matrices, respectively. The matrices were sequentially aligned in green–red–green patterns, and external magnetic bar was applied and repeatedly swiped from side-to-side. Fluorescent images were taken at 0, 10, 20, and 100 swipes, showing progressive reconfiguration of the GFP- and RFP-seeded matrices from discrete colored zones into a single region containing both cell types. Scale bar = 2 mm.

These experiments show that the combined thermoresponsive and magnetic colloid can be used not only to expand clinically important cell types such as MSCs, enabling passaging without using animal derived products such as trypsin and achieving high cell recoveries, but also to pattern cells into discrete regions and subsequently reconfigure these patterns in a noninvasive manner. The colloidal gels are formed from inexpensive and readily available materials, and are compatible with standard cell culture assays. We believe these materials should find use in manufacturing of cells through providing architectures/enviro­nments similar to those occurring in vivo, and also in tissue modeling, where specific placement of different cell types is necessary to recapitulate the cell organization of normal tissue. Further development of this technology could define a new platform 3D matrix, adaptable for a range of automated cell manufacturing, tissue modeling, and in vitro in vivo correlation assays, as desired.

## Experimental Section

*Materials*: 2-methoxyethoxy)ethyl methacrylate polymer (DD-pMEO_2_MA) and MPSMs were prepared according to literature procedures as detailed in the Supplementary Information. All other chemicals were purchased from Sigma–Aldrich or Fisher Scientific and used without further purification. All the solvents were HPLC grade, purchased from Sigma–Aldrich and used without further purification.

*Cells and Cell Culture*: Bone marrow-derived human mesenchymal stem cells (hMSCs) were purchased from Lonza. The cells were received at passage 2 and were expanded until passage 4, and were then used for experimentation. For expansion, cells were cultured in Lonza hMSC medium at 37 °C in a humidified atmosphere of 5% CO_2_. To visualize proliferation of hMSCs within the 3D matrix (Figure 3), the cells were immortalized and GFP tagged as described previously.^26^ Similarly, mouse 3T3 fibroblasts, of the NIH3T3 strains, were also modified separately to express RFP and GFP, for use in cell patterning experiments (Figure 5), as previously reported,^26^ whereas hMSCs, unlabeled, were used to investigate cell expansion, passaging, and recoveries (Figure 3 g,h and Figure 4).

*Matrix Sterilization*: The DD-pMEO_2_MA polymer was predissolved in cell culture medium (4% w/v) and filtered through a 0.2-μm sterile filter at ≈5 °C. The MPSMs were contained in a glass vial, placed on a rotating shaker, and sterilized under UV light (260-nm wavelength) for 60 min.

*Matrix Preparation*: Following the sterilization process, the matrix was prepared by mixing MPSM (25% w/v) in DD-pMEO_2_MA polymer (4% w/v) (predissolved in cell culture media). The components were thoroughly mixed and then refrigerated until further use.

*Cell Seeding into 3D Matrix*: We devised two protocols to seed cells into the 3D matrix. In the first method, cells were counted and resuspended in media (20 μL). The cells were gently mixed with the DD-pMEO_2_MA/MPSM suspension and added dropwise into prewarmed media (37 °C), instantly forming a cell-seeded 3D colloidal gel matrix. In the second method, the cells and matrix mixture were added dropwise onto the surface of PTFE-coated slides, forming a droplet that, when briefly incubated (1 min) at 37 °C, set in place to form the cell-seeded colloidal gel. This gel was subsequently transferred into prewarmed cell culture media. The two complementary methods were designed to demonstrate applicability both for research use in a lab setting and for high-throughput-automated culture formats appropriate for cell manufacture.

*Cell Viability and Proliferation*: Cell viability and proliferation were measured using a standard PrestoBlue assay according to the manufacturer's instructions (Invitrogen). Briefly, hMSCs were seeded into the 3D colloidal gel at 25 × 10^3^ cells per 25-μL volume of the matrix (formed on the PTFE surfaces as described above). To quantify the number of proliferated cells, the media were first removed from the matrix, and the cells were released from the matrix by cooling to room temperature. The cells were mixed with 10 μL of PrestoBlue reagent and incubated for 30 min. A standard calibration curve was used to quantify the total cell numbers. To draw a comparison between tissue culture plastic (TCP) and the 3D matrix, hMSCs were also seeded (25 × 10^3^ cells per well, 24 well plate) and quantified following the protocol as described above. The absorbance of the colloidal gel components (cell free) was also measured with the PrestoBlue reagent as a further control reading.

*Enzyme-Free Cell Passage*: The proliferated cells in the matrix were passaged by removing the cell culture media and cooling briefly to room temperature to allow the colloidal particles to flow and thus to release the cells. At this point, an equal volume of new colloidal particle suspension (without cells) was added to the “parent” mixture and gently mixed. Following this addition, two new cell-seeded “daughter” matrices were formed following the same protocol as described above (formed on the PTFE surfaces).

*Cell Harvesting*: Following proliferation, the cell-seeded matrices were briefly cooled to room temperature to release the cells. Subsequently, the cell and matrix mixture was drawn into a glass tube and exposed to an external magnetic field (10-mm bar magnet) in close proximity. The magnetic microparticles were separated, leaving cells only in the culture media. Finally, the cells were centrifuged at 200 *g* for 5 min to produce cell pellets and resuspended in fresh media, ready for subsequent analysis.

*Flow Cytometry*: Immunophenotypic analysis of the hMSCs was determined by flow cytometry before and after proliferation in the colloidal gel. This was performed using a BD Stemflow hMSCs analysis kit and BD LSR II flow cytometer. Cells were prepared for analysis following the manufacturer's instructions (BD-Biosciences, UK). In the case of cells proliferated in the 3D matrix, cells were first magnetically separated as described above. Mouse antihuman monoclonal antibodies CD90 FITC, CD105 PerCP-Cy5.5, and CD73 APC were used to target cell surface receptors for positive identification of hMSCs, while CD34, CD45, CD19, CD11b, and HLA-DR were used for negative expression; the antibodies were incubated with the cells in the dark at room temperature for 30 min. Associated isotype controls were also prepared for all the antibodies. A minimum of 10 000 events were recorded for each sample, and the data were analyzed using Weasel software (v3.1). Cells proliferated on TCP were also analyzed for cell surface, marker content to compare with the cells expanded in the 3D matrix.

*Controlled 3D Cell Pattern*: Cells were patterned on the 3D matrices into discrete zones. Cultured 3T3 fibroblasts expressing RFP (red) and GFP (green) were incorporated into the 3D matrices (formed on PTFE slides), using cell densities of 1 × 10^6^ per 20 μL of the colloidal particle suspension. Multiple GFP 3T3-seeded matrices, free flowing in media in a plastic Petri dish, were moved by a magnetic bar underneath to form a layer of multiple GFP-seeded matrices around a single RFP 3T3-seeded matrix in the center. Further alternating layers of RFP and GFP-seeded matrices were formed around the first layers using the same techniques. Fluorescence microscopy images were recorded at each stage to visualize the 3D cell pattern formation.

*Reconfiguration of 3D Cell Pattern in a Non-invasive Manner*: RFP 3T3 and GFP fibroblast cells were seeded into magnetic and nonmagnetic matrices, respectively (formed on PTFE slides). The matrices were aligned in such that red and green colored cells were in alternating layers, and a magnetic field bar was applied in close proximity. To reconfigure the cell patterns, the magnetic bar was swiped sequentially from side to side (1–100), and the pattern reconfiguration process was imaged using fluorescence microscopy. After reconfiguration, the cells were checked for viability using the PrestoBlue assay.
