# Supplementary material for: A Thermoresponsive and Magnetic Colloid for 3D Cell Expansion and Reconfiguration
Source: Adv Mater. 2014 Nov 29;27(4):662–8. doi: 10.1002/adma.201403626 (PMC4322481; doi:10.1002/adma.201403626)
Supplement: Supplementary file 1 [file adma0027-0662-sd1.pdf]

Copyright WILEY-VCH Verlag GmbH & Co. KGaA, 69469 Weinheim, Germany, 2014.

ADVANCED MATERIALS

Supporting Information

for *Adv. Mater.,* DOI: 10.1002/adma.201403626

A Thermoresponsive and Magnetic Colloid for 3D Cell Expansion and Reconfiguration

Aram Saeed, Nora Francini, Lisa White, James Dixon, Toby Gould, Hassan Rashidi, Racha Cheikh Al Ghanami, Veronika Hruschka, Heinz Redl, Brian R. Saunders,* Cameron Alexander,* and Kevin M. Shakesheff**

Supporting Information

A Thermoresponsive and Magnetic Colloid for 3D Cell Expansion and Reconfiguration

Aram Saeed, Nora Francini, Lisa White, James Dixon, Toby Gould, Hassan Rashidi, Racha Cheikh Al Ghanami, Veronika Hruschka, Heinz Redl, Brian R. Saunders*, Cameron Alexander*, and Kevin M. Shakesheff**

Keywords: biomaterials, thermoresponsive polymers, magnetic particles, 3D scaffolds, stem cells

Table of contents

1. .... M
aterials and Methods... 1
2. .... S
ynthesis of magnetic polystyrene microparticles (MPSM)....................................... 2
3. .... S
ynthesis of temperature sensitive polymer: DD-pMEO$_2$MA 3
4. .... M
orphology of magnetic polystyrene microparticles (MPSM) 3
5. .... M
agnetic content analysis of MPSM... 4
6. .... S
tructural analysis of DD-pMEO2MA polymer 5
7. .... D
istribution of GFP MSCs within the layers of 3D matrix... 6
8. .... P
ositive identification of BM-hMSCs with surface markers..................................... 7

1. Materials and Methods

2-(2-methoxyethoxy)ethyl methacrylate (Mn= 188), 1-dodecanethiol (98%) styrene and divinylbenzene (DVB), (80% technical grade) were purchased from Sigma-Aldrich and purified before use by passing through a column filled with neutral alumina, and stored in a refrigerator prior to use. Polyvinylpyrrolidone (PVPK-30, Mw=40 000) and 2, 2′-Azobis (2-

methylpropionitrile) (AIBN), Fe3O4 magnetic powder were purchased from Sigma-Aldrich and used without further purification.

2. Synthesis of magnetic polystyrene microparticles (MPSM)

Polystyrene microspheres were prepared by a non-aqueous dispersion polymerisation technique. In a 500 ml two neck round bottom flask equipped with a reflux condenser, ethanol (160 g), water (18 g) and PVP (3 g) were added and stirred until the PVP completely dissolved. To this mixture styrene (10 g, 96 mmoles) and DVB (0.5 g, 3.9 mmoles) were added and stirred at 100 rpm for 0.5 h with nitrogen purging. The temperature was then elevated to 74 ^{o}C and (0.4 g, 2.4 mmoles) of AIBN was added to the reaction vessel and stirred for a further 24 hours at 120 rpm. The polystyrene microspheres were separated by centrifugation (3000x g for 10 minutes) and repeatedly washed by re-suspension and centrifugation steps with distilled water. The resulting microspheres were dried in vacuum oven, at 60 ^{o}C overnight.

The dried microparticles were further used as a core and an extra layer of styrene containing Fe_3O_4 was polymerised on the surface. In a round bottomed flask, a suspension of 4% (w/v) microparticles were added under mechanical stirring. The reaction mixture was sealed with a rubber septum and purged with nitrogen for 30 minutes. The reaction temperature was increased to 60 ^{o}C. At this point, a mixture of styrene 6 ml and 20 % (w/w) Fe_3O_4 (compared to the amount of the microparticles in the flask) were added. The reaction mixture was stirred for further 6 hours. At this point, 6 ml of styrene was added and the reaction left to stir overnight before purification. The mixture was purified by passing through double-layered cloth, followed by magnetic decantation and washing with distilled water several times. Finally, the obtained magnetic microparticles were dried in a vacuum oven at 40 ^{o}C for 24 hours.

3. Synthesis of temperature sensitive polymer: DD-pMEO2MA

2-(2-methoxyethoxy)ethyl methacrylate MEO2MA (10 g Mn= 188, 54 mmoles), 1-dodecanethiol (0.08 g, 0.40 mmoles) and AIBN(0.065g, 0.40 mmoles) were added into a 100 ml round bottomed flask containing 25 ml of butanone. The reaction mixture was degassed with nitrogen for 15 minutes before the contents of the flask were heated to 70 oC. After two hours of polymerisation, the reaction was stopped by cooling and the polymer precipitated in an excess of hexane to remove unreacted monomers.

4. Figure S1. Morphology of magnetic polystyrene microparticles (MPSM)

Representative scanning electron microscope of magnetic polystyrene microparticles. Scale bar = 10 µm

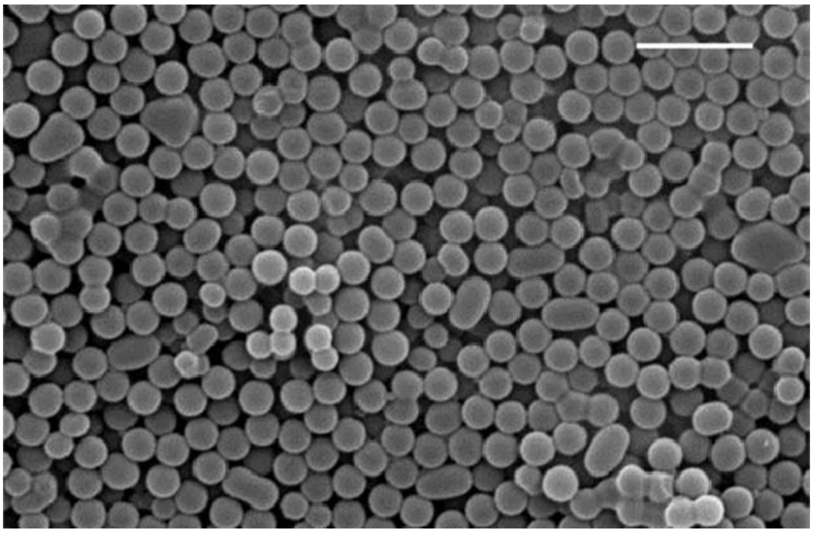

5. **Figure S2. Magnetic content analysis of MPSM**

Representative thermogravitational analysis (TGA) of plain polystyrene microparticles (blue line) and magnetic polystyrene microparticles (red line). As can be seen from the weight loss, the 15.4% weight remaining in the MSPPM at > 400 °C was attributed to the Fe_3O_4 content.

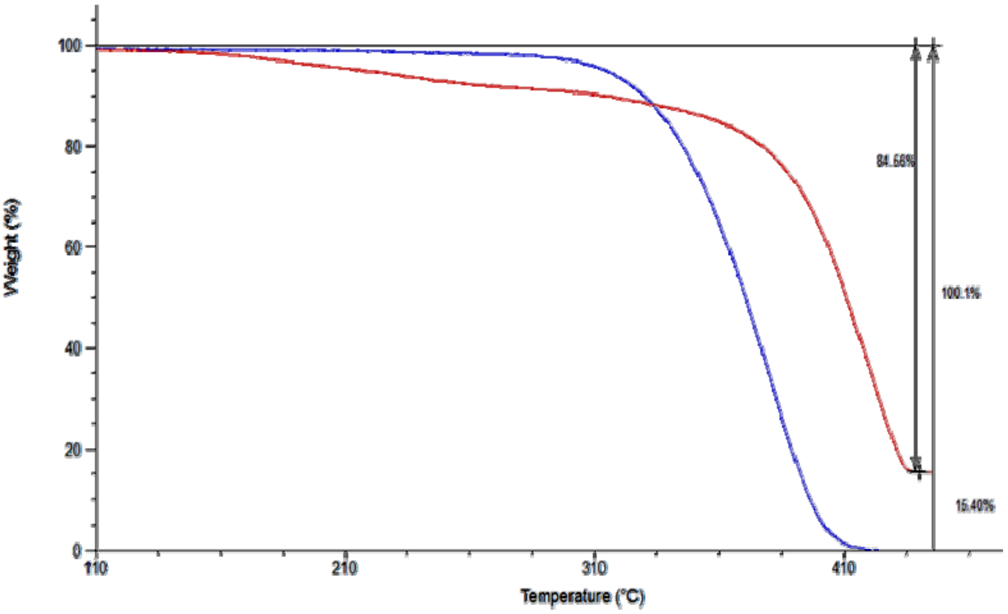

6. Figure S3. Structural analysis of DD-pMEO2MA polymer

Representative ^{1}HNMR spectra of D-pMEO_2MA temperature sensitive polymer, relevant signals integrated on the polymer.

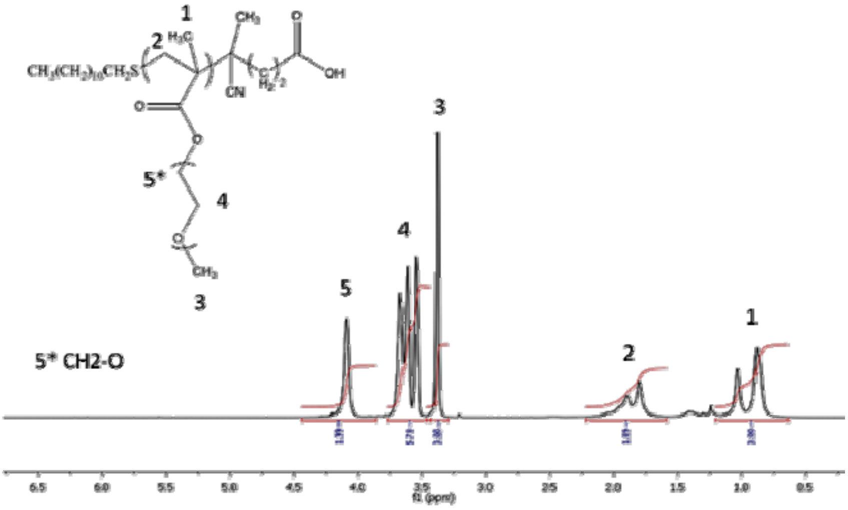

7. **Figure S4. Distribution of GFP MSCs within the layers of 3D matrix**

Representative examples of homogenous cell growth of GFP-MSCs throughout the 3D scaffold at day 16, sectioned and divided into top, middle and bottom layer. Scale bar = 100 μm

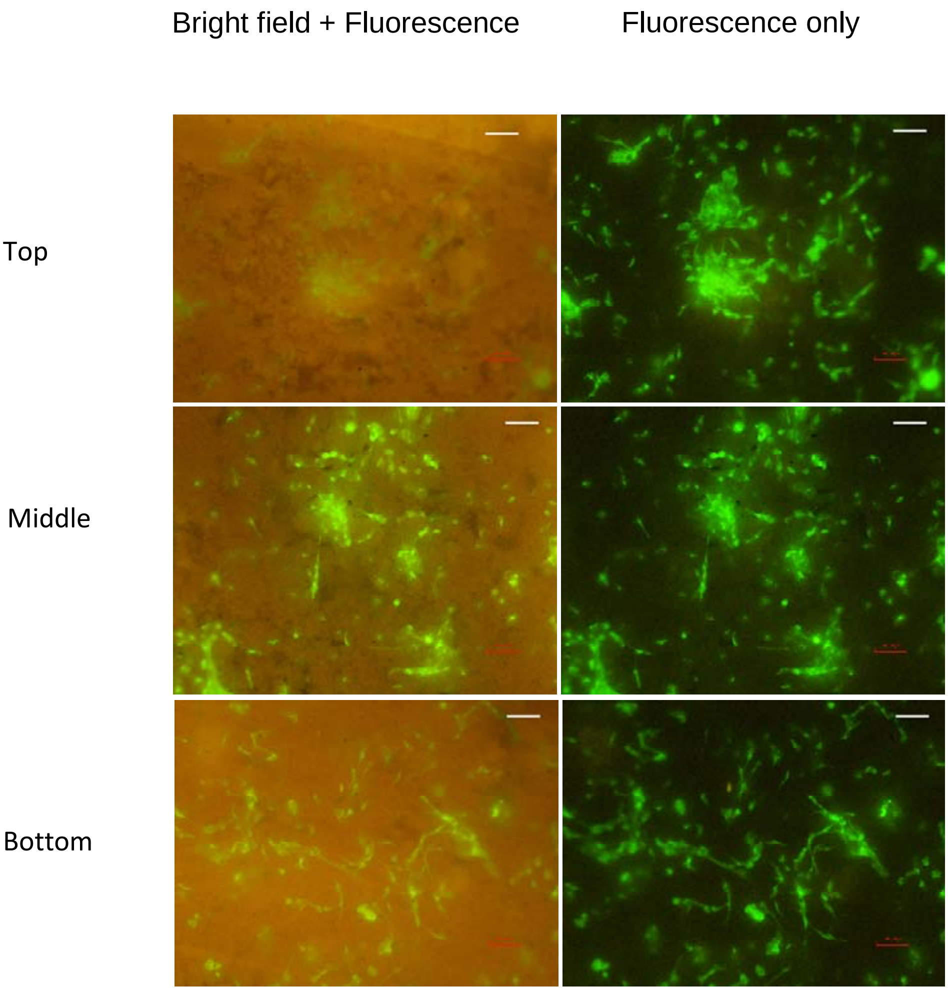

8. **Table S1. Immunophenotypic expression of hMSCs before and after cell culture on 2D TCP and 3D matrix.** Using human MSC analysis kit (BD Stemflow™) the hMSCs were analysed for positive CD73, CD90, and CD105, and negative CD34, CD45, CD19, CD11b and HLA-DR makers. Flow cytometry data were collected using BD LSR II and analysed using Weasel flow software V3.1.

	Pre-inoculation (Day 0)	Post-inoculation 2D (Day 5)	Post-inoculation 3D (Day 5)
**CD90 (+ve)**	99.9 ± 0.05	99.7 ± 0.15	99.1 ± 0.25
**CD73 (+ve)**	99.9 ± 0.057	99.8 ± 0.11	99.3 ± 0.15
**CD105 (+ve)**	99.8 ± 0.11	99.7 ± 0.15	99.4 ± 0.10
**CD11b (-ve)**	0.18 ± 0.09	0.48 ± 0.10	0.37 ± 0.18
**CD19 (-ve)**	0.35 ± 0.18	0.40 ± 0.049	0.40 ± 0.017
**CD34 (-ve)**	0.18 ± 0.09	0.35 ± 0.06	0.42 ± 0.08
**CD45 (-ve)**	0.26 ± 0.18	0.39 ± 0.07	0.37 ± 0.13
**HLA-DR (-ve)**	0.09 ± 0.04	0.30 ± 0.45	0.72 ± 0.20
